# Spatial Variations of the Methanogenic Communities in the Sediments of Tropical Mangroves

**DOI:** 10.1371/journal.pone.0161065

**Published:** 2016-09-29

**Authors:** Hongmei Jing, Shunyan Cheung, Zhi Zhou, Chen Wu, Sanjay Nagarajan, Hongbin Liu

**Affiliations:** 1 Sanya Institute of Deep-sea Science and Engineering, Chinese Academy of Sciences, Sanya, China; 2 Division of Life Science, The Hong Kong University of Science and Technology, Clear Water Bay, Kowloon, Hong Kong SAR, China; 3 Department of Civil and Environmental Engineering, Faculty of Engineering, National University of Singapore, Singapore, Singapore; 4 Division of Environmental and Ecological Engineering and School of Civil Engineering, Purdue University, West Lafayette, Indiana, United States of America; CAS, CHINA

## Abstract

Methane production by methanogens in mangrove sediments is known to contribute significantly to global warming, but studies on the shift of methanogenic community in response to anthropogenic contaminations were still limited. In this study, the effect of anthropogenic activities in the mangrove sediments along the north and south coastlines of Singapore were investigated by pyrosequencing of the *mcrA* gene. Our results showed that hydrogenotrophic, acetoclastic and methylotrophic methanogens coexist in the sediments. The predominance of the methylotrophic *Methanosarcinales* reflects the potential for high methane production as well as the possible availability of low acetate and high methylated C-1 compounds as substrates. A decline in the number of acetoclastic/methylotrophic methanogens in favor of hydrogenotrophic methanogens was observed along a vertical profile in Sungei Changi, which was contaminated by heavy metals. The diversity of methanogens in the various contaminated stations was significantly different from that in a pristine St. John’s Island. The spatial variation in the methanogenic communities among the different stations was more distinct than those along the vertical profiles at each station. We suggest that the overall heterogeneity of the methanogenic communities residing in the tropical mangrove sediments might be due to the accumulated effects of temperature and concentrations of nitrate, cobalt, and nickel.

## Introduction

Methane (CH_4_) is a key component in the global carbon cycle. As a major green-house gas, it is approximately 26 times more effective than CO_2_ in retaining heat in the atmosphere [1]. The atmospheric CH_4_ inventory is currently increasing by ~0.4% per year [2]. Mangrove wetlands and paddy fields, as well as the enteric fermentation that occurs during digestion in ruminants are the most important sources of atmospheric CH_4_ [3,4]. Among them, the mangrove wetlands are the largest natural source of CH_4_, contributing about 20% of the total annual emission to the atmosphere [5,6].

The mangrove wetlands are very productive coastal ecosystems and various anaerobic microbial processes occur in their predominantly anoxic sediments. In these sediments, CH_4_ is produced during the terminal stage of anaerobic decomposition of organic matter by methanogens [7], when the redox potential of the sediment reached to below -150 mV [8]. Methanogens are strictly anaerobic archaea and so they are very sensitive to of O_2_ [9]. The onset of methanogenesis primarily occurs at a shallow depth (i.e., 20–25 cm) of the sediments. The CH_4_ produced undergoes vertical diffusive transportation from the sediment surface to the atmosphere, and horizontal transportation to the adjacent estuarine and coastal water column [10]. Natural factors such as the temperature, salinity and organic carbon content of the sediment [11] have also been shown to affect the geographical variation in the production and emission of CH_4_ in mangrove wetlands. In addition, several anthropogenic factors, such as disposal of sewage and agricultural runoff into the mangrove ecosystem have also been reported to enhance the emission of CH_4_ [12].

Methanogens belong to the *Euryarchaeota* phylum of the Archaea domain, and consist of six phylogenetically diverse orders, *Methanobacteriales*, *Methanococcales*, *Methanomicrobiales*, *Methanocellales*, *Methanopyrales* and *Methanosartinales*, and 33 genera based on the gene sequences of 16S rRNA [13,14]. Methanogens are widely distributed in natural, strictly anaerobic environments, such as: flooded rice fields [15]; freshwater and marine sediments [16,17]; deep-sea hydrothermal vents [18,19]; marine mud volcanoes [20]; hot springs [21]; and mangroves [22]. By far, most studies on methanogens in mangrove sediments were focused on the tropical regions. For example, *Methanococcoides* were important component in the Tanzanian mangrove [23] and *Methanomicrobia* and *Methanobacteria* were the two most abundant groups in the sediments of Sundarbans in India [24]; while *Methanomicrobia* dominated in the sediments of Guanabara Bay [25] and Sao Paulo state in Brazil [26]. In a recent study on the subtropical mangrove in Mai Po in China, groups of Methanomicrobiales, Methanosarcinales and Methanobacteriales were revealed [27]. However, knowledge about the phylogenetic composition of methanogens has until recently been limited by the traditional culture-based procedures and conventional molecular techniques [28]. The recently-developed pyrosequencing technology might significantly enhance the detection capability of rare species, and when applied together with the functional *mcrA* gene, the complex methanogenic communities in natural anaerobic environments might be more accurately defined [29,30]. The *mcrA* gene, which is unique to and ubiquitous among all known methanogens [31], encodes the α-subunit of methyl coenzyme M reductase, which is the terminal enzyme involved in the methanogenesis pathway, where methane is released [31].

**Fig 1:**
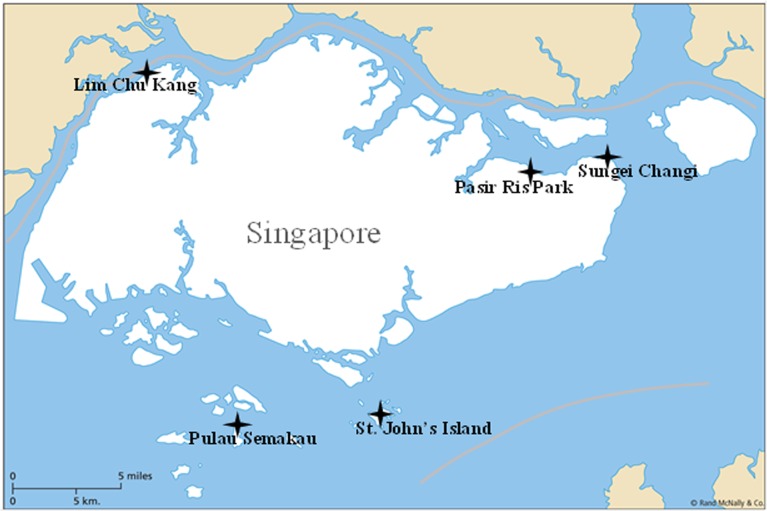
The five mangrove sampling stations located along the Singapore coastline. Fig 1 was modified from a free picture from Wikipedia. Wikipedia has a free license "Permission is granted to copy, distribute and/or modify this document under the terms of the GNU Free Documentation License, Version 1.2 or any later version published by the Free Software Foundation; with no Invariant Sections, no Front-Cover Texts, and no Back-Cover Texts. Subject to disclaimers".

The Singapore coastline harbors extensive areas of mangrove wetlands, but these ecosystems have suffered from both natural and anthropogenic disturbances in recent years following the increase in population and consequent industrialization. It is thought that the increased input of external nutrients and metals into the mangrove sediments from the adjacent areas might cause significant variations in the composition and activity of different microbial communities, especially methanogens. In order to better understand the anthropogenic and ecological impact on the methanogenic population in the tropical mangrove, sediment samples were collected from five tropical mangroves along the north and south coast of Singapore. These were Lim Chu Kang (LCK), Pulau Semakau (PS), Sungei Changi (SC), Pasir Ris Park (PRP) and St. John’s Island (SJ) (Fig 1). LCK is characterized by its strong agriculture activities; PS is the site of a new landfill; PRP is the location of the first toxic algal bloom in Singapore, which occurred in 2009, and it was shown to contain high levels of total nitrogen during our sampling in 2012; SC is near to Changi airport and is downstream of both PRP and an old landfill site located at Sungei Punggol; and SJ, which is located far from any industrial or residential areas, was considered to be a pristine location [32]. In this study, pyrosequencing of the functional *mcrA* gene, which is a biomarker of methanogens, was applied to investigate the methanogenic populations residing in the tropical mangrove sediments in these various geographical conditions and subjected to different anthropogenic perturbations, and to elucidate the key environmental impact factors.

## Materials and Methods

### Sample collection and biogeochemical analysis

In October 2012, mud samples were collected in triplicate from five mangrove locations along the coastline of Singapore with research permit issued from the National Parks Board in Singapore (Fig 1). At each location, approximately 50 g sediment at three depths (i.e., 1–2 cm (shallow), 10–11 cm (middle) and 20–21 cm (deep)), from the surface, were collected and placed in 15 ml Falcon tubes. They were kept on ice in the field, and then stored at -80^°^C prior to further analysis.

At each sampling station, various *in situ* environmental parameters (e.g., location, temperature, salinity, and pH) were recorded during field sampling. In addition, the levels of both nutrients (i.e., total phosphate (TP), total inorganic nitrogen (TIN, including NH_4_^+^, NO_3_^-^ and NO_2_^-^), and heavy metals (i.e., As, Ba, Co, Cr, Ga, Li, Ni and Pb) were measured, as described previously [32]. The biogeochemical characteristics of the five locations are summarized in Tables 1 and 2.

**Table 1:** The environmental parameters at the different locations used in this study^§^.

Locations	pH	Temp (°C)	Moisture (%)	NO_3_^-^ (μg/g)	NO_2_^-^ (μg/g)	NH_4_^+^ (μg/g)	TIN (μg/g)	TP (μg/g)
**LCK_S**	6.69±0.07	27.4±0.0	30.53±1.84	62.11±1.51	2.38±0.04	1.40±0.25	65.89±1.72	36.69±5.80
**LCK_M**	6.35±0.12	27.3±0.0	40.79±2.24	65.57±3.98	2.45±0.15	0.41±0.08	68.42±4.19	7.49±0.00
**LCK_D**	6.40±0.10	27.0±0.0	41.37±4.01	63.22±2.29	2.43±0.09	0.78±0.03	66.42±2.39	-
**PRP_S**	7.31±0.07	26.5±0.0	30.21±1.76	56.15±0.12	2.32±0.03	0.78±0.02	59.25±0.15	74.39±6.81
**PRP_D**	8.61±0.09	27.2±0.0	24.26±0.18	58.03±0.12	2.42±0.02	1.26±0.01	65.05±3.34	10.58±1.33
**PS_M**	7.59±0.01	28.7±0.0	30.49±1.72	53.03±1.26	2.64±0.10	1.04±0.01	56.71±1.38	129.12±2.86
**SC_S**	7.27±0.03	25.0±0.0	14.75±0.44	56.46±1.69	2.47±0.07	1.20±0.02	60.13±1.73	-
**SC_M**	7.02±0.08	24.0±0.0	17.77±1.04	68.34±1.92	2.53±0.09	1.78±0.10	60.49±2.10	-
**SC_D**	6.37±0.23	21.0±0.0	16.18±0.37	50.56±1.85	2.55±0.18	0.41±0.03	53.52±2.03	-
**SJ_S**	7.00±0.08	27.8±0.0	23.24±0.86	48.01±1.98	2.33±0.10	1.00±0.02	51.35±2.06	57.44±12.42
**SJ_M**	7.05±0.01	28.2±0.0	24.52±0.24	52.57±0.49	2.55±0.05	1.66±0.08	56.78±0.58	20.30±7.66
**SJ_D**	7.10±0.02	28.3±0.0	22.18±4.16	46.61±1.50	2.41±0.08	0.31±0.12	49.33±1.70	42.83±0.00

Note:

^§^All the data in this table have been cited in Xia *et al*, under preparation.

**Table 2:** The concentrations of the various metals at the different locations in this stud^§^.

Locations	Cr	Co	Ni	Ga	Pb	As	Ba	Li
**LCK_S**	8.91±0.41	0.27±0.13	2.28±0.12	5.98±0.32	6.41±1.35	28.19±3.27	6.75±0.62	16.86±0.52
**LCK_M**	12.78±0.07	0.50±0.50	3.36±0.37	7.10±0.39	9.98±0.53	48.19±10.78	4.43±0.18	20.19±0.79
**LCK_D**	13.46±1.26	1.89±0.26	4.16±0.50	7.81±0.09	12.82±0.22	78.56±12.68	3.85±0.62	22.52±0.25
**PRP_S**	6.78±0.49	4.08±0.40	4.55±0.54	7.41±0.06	14.00±0.93	5.25±0.35	68.22±0.82	4.69±0.21
**PRP_D**	3.91±0.28	4.29±0.11	3.91±0.10	6.54±0.12	10.09±0.33	2.37±0.83	55.86±3.40	2.90±0.24
**PS_M**	9.26±0.37	2.70±0.03	7.05±0.51	7.97±0.62	7.53±0.86	16.64±0.24	5.27±0.56	10.27±0.79
**SC_S**	49.08±1.45	37.08±0.57	46.71±0.56	10.98±0.43	433.87±14.45	36.95±0.68	88.69±4.32	10.81±0.86
**SC_M**	45.85±1.55	35.39±0.45	44.10±0.69	9.46±0.47	115.95±7.88	36.22±0.52	60.40±1.56	13.46±0.92
**SC_D**	49.26±1.37	35.19±0.51	44.71±0.44	10.46±0.75	79.29±2.41	37.90±1.19	60.40±1.78	17.23±0.10
**SJ_S**	10.46±1.33	1.58±0.39	4.07±0.69	5.92±0.37	9.26±0.26	1.78±0.19	14.62±1.26	4.74±0.02
**SJ_M**	5.96±0.16	1.23±0.01	3.49±0.18	4.11±0.38	15.16±1.70	2.26±0.63	4.93±0.37	3.08±0.18
**SJ_D**	5.26±0.05	0.78±0.02	2.93±0.28	3.90±0.69	14.03±2.41	1.94±0.34	6.19±1.39	3.11±0.30

Note

^§^All data in this table have been cited from Xia *et al*, under preparation; Unit of all metals (μg/g).

### 454 Pyrosequencing and bioinformatics analysis

Genomic DNA from three independent samples was extracted and pooled together (~250 mg) as templates for amplification of the *mcrA* gene. Amplification was conducted with degenerate ML primers using the PCR protocol described by Luton *et al*. [33]. In order to enable sample multiplexing during sequencing, barcodes were incorporated between the adapter and forward primer. Nuclease-free water was used as the negative control in each reaction. Triplicate PCRs were performed for each sample and the amplicons were pooled together for subsequent purification, amplicon library construction and pyrosequencing, as described previously [32].

Raw sequence data were processed using the microbial ecology community software program Mothur [34]. Low quality sequences (with an average quality score < 25), short sequences (< 350 bases in length), ambiguous base-containing sequences, homopolymer-containing sequences (> 8 bases), chimeric sequences, and barcodes of the sequences were removed. The trimmed sequences were de-noised with 0.01 sigma value to reduce possible effects of PCR bias, after which the sequences were aligned with the reference sequences of the *mcrA* gene from the National Center for Biotechnology Information (NCBI; http://www.ncbi.nlm.nih.gov/). The quality reads generated from the three samples, PRP_M, PS_S and PS_D, were less than 1000, therefore, they were not included for the analysis to avoid bias. The remaining quality sequences were then used to define the operational taxonomic units (OTU) with 97%, 89% and 79% sequence similarity, as cutoff values to represent the species, genus and family levels, respectively [35]. The OTUs that contain only one sequence were removed. The richness estimator (Chao1), diversity (Shannon-Weaver index, *H*'), and Good’s coverage were calculated with three cutoff values after sequence normalization. This resulted in an equal number of sequences for each sample by randomly selecting within each sample according to the sample with the least number of sequences. In addition, a rarefaction curve was generated with a 97% sequence similarity as the cutoff value. To identify the phylogenetic affiliation of the *mcrA* sequences, representative sequences of the 50 most abundant OTUs (with 97% cutoff value) were used to search the nucleotide BLAST (BLASTn) webpage of the NCBI nucleotide sequence database (http://blast.ncbi.nlm.nih.gov/Blast.cgi). The representative sequences of the top 50 OTUs, the selected reference sequences and the environmental sequences of the *mcrA* gene from the NCBI database were used to construct a neighbor-joining (NJ) tree using the MEGA 6.0 (molecular evolutionary genetics analysis) software [36]. To evaluate the number of shared OTUs (with 97% cutoff) among samples, normalized OTU data were also used for generating a Venn diagram using R [37].

### Statistical analysis

To assess the dissimilarity among multiple groups, a newick-formatted tree was generated using the tree.shared command in Mothur. In the same software, the Thetayc calculator was used to determine the UPGMA (unweighted pair group method with arithmetic mean) clustering at genus level (with 89% cutoff value). In addition, a redundancy analysis (RDA) was performed using CANOCO V4.5, to reveal relationships between the structure of the various methanogenic communities (with 97% cutoff value) and environmental variables [38]. All the data were root-square transformed and the effects of high collinearity among factors were removed. Forward selection was used to determine the minimum set of environmental variables that might explain the largest amount of variance in the microbial community. The statistical significance of an explanatory variable added in the course of forward selection was tested with the Monte Carlo permutation test (999 permutations, *p* < 0.05). For all community ordination analyses, biplot scaling was used.

### Accession number

All the *mcrA* sequences obtained from this study were deposited in the NCBI Sequence Read Archive (SRA) under the accession number of SRP068266.

## Results and Discussion

### Sampling locations and diversity of methanogens

Of the five sampling locations selected, SJ was the least influenced by human activities. We therefore used this pristine location on the southern coastline of Singapore as the background as we have done in a previous study [32]. SJ contained the lowest concentration of dissolved inorganic nitrogen (DIN), when compared with the other locations. The old landfill site at PS is also located on the southern coastline. This had a similar temperature as SJ, but exhibited much higher levels of TP (Table 1). Sediment in LCK was acidic and had the highest content of TIN as a result of strong agricultural activity. Relatively high concentrations of TIN (especially NO_3_^-^) and TP were also detected in PRP, where a toxic algal bloom had occurred near its maritime space in December 2009. SC is adjacent to Changi airport and had the highest content of most of the heavy metals measured (i.e., Cr, Co, Ni, Ga, Pb, and Ba) (Table 2), but the lowest temperature, moisture and conductivity (Table 1). In general, the surface sediment at SC (SC_S) had the highest content of TP, but there was no obvious depth profile for the other parameters listed in Table 1. With regards to the depth profiles in the other locations; in LCK, all the metals except Ba, exhibited a depth-wise incremental increase; whereas in SJ, four metals, (i.e., Cr, Co, Ni and Ga), exhibited a depth-wise decrease (Table 2).

**Fig 2:**
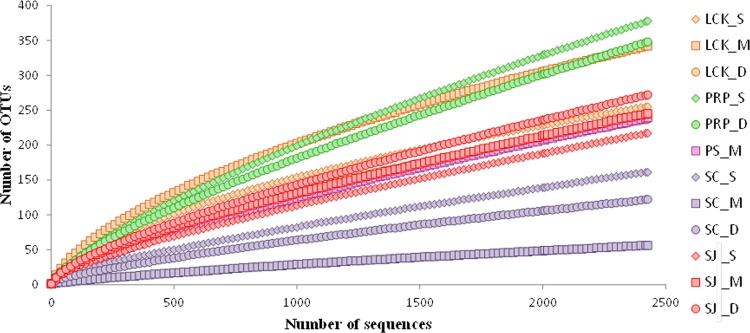
Rarefaction curves for the *mcrA* gene sequences obtained by amplicon pyrosequencing from samples collected from the five locations in Singapore with 97% sequence similarity as the cutoff value.

Pyrosequencing generated on average 3,742 quality reads per sample (Table 3), after the low quality reads were filtered out according to the criteria described in the Materials and Methods. At the species level (97%), the highest and lowest numbers of OTUs were found in PRP and SC, respectively (Table 3). In the locations affected by anthropogenic activities (i.e., LCK, PRP, SC), a higher diversity of methanogens was observed in the shallow layer than in the deep layer. This is in agreement with previous reports, which showed that a higher methanogenic diversity occurs in the shallow layers of sediments [19,29], possibly as a result of organic enrichment in the surface sediment. In contrast, in the pristine SJ, the highest diversity and number of OTUs along with the lowest concentrations of inorganic nitrogen were found in the deepest layer (SJ_D). When compared with the pristine SJ, the diversity was higher in both LCK and PRP, which contained higher nutrient levels, and it was lower in SC, which was contaminated with high levels of heavy metals (Table 3). Our results therefore showed that the diversity of methanogens was significantly different among the different locations, and we suggest that the differences observed along the vertical profiles might be explained by the *in situ* substrate composition and anaerobic conditions in the tropical mangroves. The diversity of our samples was generally higher than those in subtropical [27] and tropical [24] mangrove sediments, although the subtropical study was also investigated by 454 pyrosequencing. The coverage at the species level was more than 89%, which is consistent with the tendency of rarefaction curves (Fig 2). This indicates that sufficient sampling efforts were applied in this study to allow for the adequate assessment of the microbial community composition in each sample.

**Table 3:** The sequencing statistics and diversity estimates for the samples collected from the different locations in this study.

Locations	High Quality Reads	Average Length (bp)	97%				89%				79%			
			OTU	Chao	*H'*	Coverage	OTU	Chao	*H'*	Coverage	OTU	Chao	*H'*	Coverage
**LCK_S**	4,613	464	255	534.3	3.5	0.96	116	245.00	2.0	0.98	60	176.6	1.0	0.99
**LCK_M**	4,527	463	342	903.6	4.0	0.94	148	339.90	2.6	0.97	76	113.6	1.5	0.99
**LCK_D**	4,502	465	237	705.1	3.3	0.95	123	379.80	1.7	0.97	68	214.4	0.9	0.99
**PRP_S**	2,706	464	378	1291.4	3.6	0.89	176	402.10	2.4	0.96	93	210.2	1.3	0.98
**PRP_D**	2,563	463	348	1590.9	3.4	0.89	156	359.30	2.3	0.96	88	181.0	1.6	0.98
**PS_M**	3,672	466	239	989.1	2.1	0.93	100	293.50	0.8	0.97	49	314.0	0.6	0.99
**SC_S**	4,238	457	161	693.1	2.7	0.96	87	200.30	1.9	0.98	55	129.4	1.8	0.99
**SC_M**	4,108	467	56	156.6	0.4	0.98	31	140.30	0.2	0.99	19	32.8	0.2	1.00
**SC_D**	4,429	443	122	374.8	1.7	0.97	49	147.20	1.1	0.99	29	43.2	1.0	1.00
**SJ_S**	2,428	463	217	1245.2	2.7	0.93	101	409.30	1.0	0.97	58	191.2	0.8	0.98
**SJ_M**	3,542	465	245	798.0	2.7	0.93	94	224.60	0.9	0.98	48	67.5	0.7	0.99
**SJ_D**	3,576	466	272	1264.5	3.0	0.92	110	425.40	1.0	0.97	65	475.0	0.7	0.98

Note: 97%, 89% and 79% cutoff values were applied for the respective species, genus and family levels [30].

### Phylogeny of methanogens

**Fig 3:**
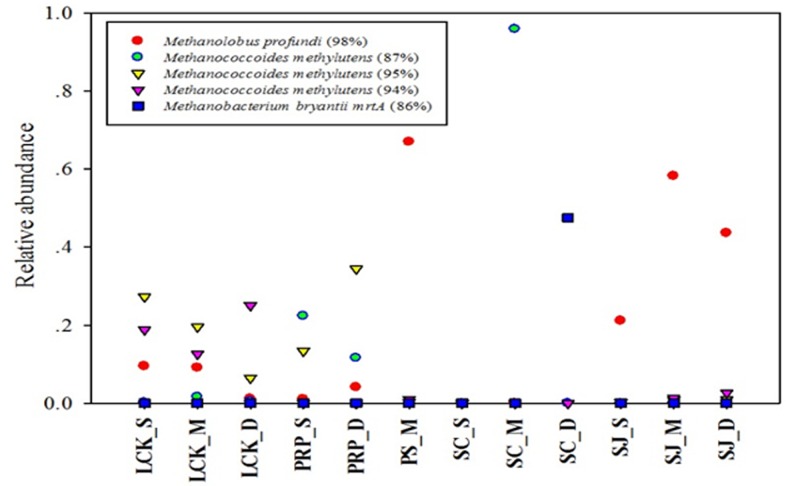
Identity and distribution of the five most abundant OTUs (with 97% sequence similarity as the cutoff value) among all the samples collected from the five locations in Singapore.

The spatial distribution of the five most abundant OTUs based on the total reads obtained, was highly variable among the different samples (Fig 3). OTU1 was the most abundant, and showed close affiliation with *Methanolobus profundi*. It was found in all the samples except SC and it accounted for high proportions in the middle depths of PS and SJ. *M*. *profundi* is a member of the mesophilic methylotrophic *Methanosarcinaceae* family and it has previously been isolated from deep subsurface sediments [39]. OTU2, 3 and 4 were all identified as *Methanococcoides methylutens* but with different similarity levels (Fig 3). The highest proportion of OTU2 (87% similarity) was found in SC_M, whereas no OTU3 (95% similarity) or OTU4 (94% similarity) were found in this station. *M*. *methylutens* has been reported to be an important methanogenic methylotroph in tropical mangrove sediments [23], where it utilizes trimethylamine, diethylamine, monomethylamine, and methanol as substrates for growth and methanogenesis [40]. The capability of methylotrophic methanogens to utilize noncompetitive substrates such as methanol, mono-, di- and trimethyl-amines, which are not easily used by sulfate reducing bacteria (SRB) [41], helps the two to co-exist in anoxic sediments. As OTU1-4 are all methylotrophic methanogens, this indicates that the substrates are available as well as the major role played by the methane production pathway in the tropical mangrove sediments.

OTU5 was identified as *Methanobacterium bryantii* mrtA with low similarity (86%), and it was present with high abundance only in SC_D (47.52%). *M*. *bryantii* is a hydrogenotrophic methanogen, which utilizes H_2_ as the sole energy substrate, and NH_4_^+^ as the essential nitrogen source [42]. The *mcrA* gene is unique to all methanogens [43], however, for members of the *Methanobacteriales* and *Methanococcales* orders, *mrtA* encoding an isoenzyme of *mcrA* is carried additionally [44]. The degenerate ML primers that were used in this study might facilitate the detection of a wide range of *mcrA* genes in environmental samples, but they might also amplify the methanobacterial *mrtA* gene [33]. Therefore, the assessment of the methanogenic community composition and abundance in environmental samples should be interpreted carefully, if based on the relative *mcrA/mrtA* gene frequencies using the ML primers exclusively. Isolating pure cultures and incorporating primers specific to the *mrtA* gene [35], would help to determine if the *mcrA* and *mrtA* sequences originate from the same species or not.

**Fig 4:**
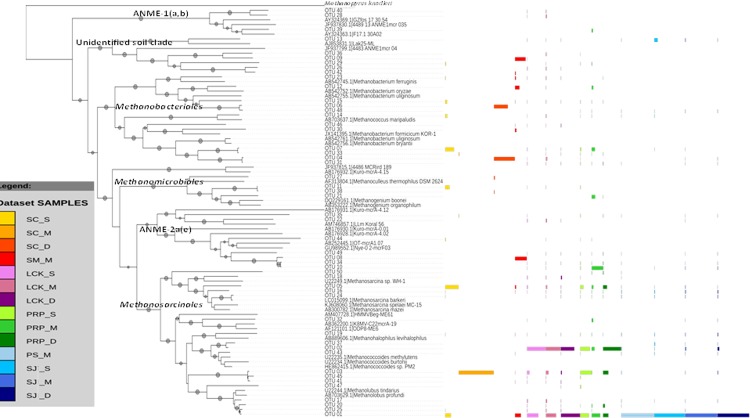
Neighbor-joining phylogenetic tree illustrating the 50 most abundant OTUs (with 97% sequence similarity as the cutoff value) among all the samples collected from the five locations in Singapore. A bootstrap value greater than 50% is shown (calculated 1,000 times). The color scale indicates the OTU distribution in the different locations.

Methanogens are phylogenetically and ecologically diverse Euryarchaeota. Phylogenetic trees based on the *mcrA* gene constructed using neighbor-joining (NJ) and maximum-likelihood methods have a congruent tree topology. We showed that the 50 most abundant OTUs at the species level in the NJ tree fell into six clades (Fig 4), including four methanogenic clades and two methanotrophic clades. In the *Methanosarcinales* clade, all 18 OTUs were closely related to *Methanosarcina*, *Methanococcoides*, *Methanolubus* and *Methanohalophilus*; in the *Methanomicrobiales* clade, four OTUs were affiliated with *Methanoculleus* and *Methanogenium*; in the *Methanobacteriales* clade, 12 OTUs were clustered with *Methanobacterium* and *Methanococcus*; and the remaining six OTUs were grouped with methanogenic sequences from soil. Two groups of anaerobic methanotrophic archaea, (i.e., ANME-1(a,b) (3 OTUs) and ANME-2a(e) (7 OTUs)), were also revealed, because *mcrA* is a phylogenetically conserved gene in both the methanogenic and methanotrophic archaea [43]. These two subgroups have different niche preferences; subgroup ANME-1 usually dominates in sulfate-depleted sediments and forms a discrete phylogenetic group; whereas subgroup ANME-2 dominates in shallow sediments containing relatively higher sulfate concentrations, and is closely related to *Methanosarcinales* [45]. Methanotrophs might eliminate methane fluxes from sediments into the overlying water column and atmosphere, and are critical for regulating the global carbon fluxes.

### Community composition of methanogens

**Fig 5:**
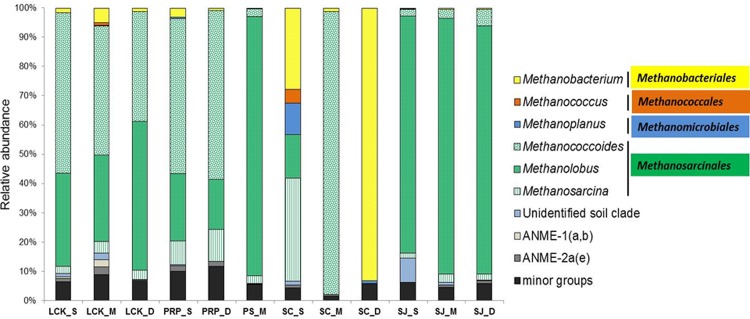
Community composition of methanogens at the genus level for samples collected from the five locations in Singapore. Phylogenetic groups accounting for less than 2% of the total community in each location were treated together as a minor group.

In total, four orders of methanogens were identified from all the samples, including hydrogenotrophic *Methanobacteriales*, *Methanococcales*, *Methanomicrobiales* and potentially acetoclastic/methylotrophic *Methanosarcinales* (Fig 5). *Methanosarcinales* predominated in almost all our samples; this is in contrasting to the fact that *Methanomicrobacteriales* and *Methanomicrobiales* were found as major methanogenic groups in the tropical mangroves in India [24] and Brazil [25]. In addition, no *Methanococcales* was detected in a recent study on a subtropical mangrove in Asia [27]. These different reports highlighted the highly diverse methanogenic communities in our sampling sites. Although *Methanoculleus*, *Methanogenium* of the *Methanomicrobiales* and *Methanohalophilus* of the *Methanosarcinales* were also detected in our study, each accounted for less than 2% of the total community. They were therefore grouped together and defined as a minor group. *Methanobacteriales*, *Methanococcales* and *Methanomicrobiales* produce methane via the reduction of CO_2_ with hydrogen gas (i.e., hydrogenotrophic methanogenesis) [46]. Only one genus was found for each hydrogenotrophic order. *Methanobacteriales* were present in all the samples, and some sequences were classified as *Methanobacterium* mrtA. *Methanoplanus*, a genus of the *Methanomicrobiales*, was absent from PS and SJ. In general, hydrogenotrophic methanogens accounted for less than 10% in all our samples, except at SC_S. This indicates that hydrogenotrophic methanogenesis is not an important pathway for methane production in the tropical mangrove sediments we studied.

In our study, the *Methanosarcinales* comprised of *Methanococcoides*, *Methanolobus* and *Methanosarcina*, which all belong to the *Methanosarcinaceae* family. This is a highly diversified group in terms of the substrates used for methane production; they are capable of growing on acetate, methanol, methylamines and H_2_/CO_2_ [47], and they play a primary role in the conversion of acetate to methane. In SJ and PS, methanogens of the genus *Methanolobus* were predominant, but in PRP *Methanolobus* was exceeded by *Methanococcoides*; and in LCK, both were major groups. In SC, *Methanosarcina* accounted for 35.25% in SC_S, whereas SC_M was exclusively composed of *Methanococcoides* (Fig 5). *Methanolobus* and *Methanococcoides* are methylotrophic methanogens, which grow entirely on methylated compounds for their nutrient source [40]. *Methanosarcina* are the only known methanogens that produce methane using all three metabolic pathways (i.e., the hydrogenotrophic, acetoclastic and methylotrophic pathways), and they are also known to tolerate oxygenated conditions [48]. In SC, there was a decline in the number of potential acetoclastic/methylotrophic methanogens in favor of hydrogenotrophic methanogens along the vertical profile; and at the deepest level of SC (SC_D), the hydrogenotrophic *Methanobacteriales* (93.07%) were predominant and no *Methanosarcinales* were present. It is well known that the availability of substrate (as well as other environmental parameters) may result in the selective proliferation of some methanogenic groups, and this eventually determines the composition of the communities that form. The variation in the methanogenic groups that exist along the vertical profile in SC might be due to the distinct physio-chemical characteristics of the sediments at the different depths. It is not surprising to find hydrogenotrophic methanogens in the deepest layer in SC (SC_D). This is because they compete with SRB for substrates in the shallow layers but this competition doesn’t exist in the deeper sulfate-depleted sediments. In contrast, methylotrophic methanogens avoid direct competition with SRB and so they are known to survive in marine sediments and in the shallower sediments in estuaries [49,50]. All the samples, except those obtained from SC_D, were predominated by the methylotrophic *Methanococcoides* and *Methanolobus*, suggesting that methane production in the mangrove sediments occurred via methylotrophic pathways. In addition, the overall predominance of *Methanosarcinales* along the vertical depth profile in our study might be attributed to their tolerance to the low levels of oxygen [47].

Methanogens utilizing different substrates for methanogenesis have been reported to coexist in various anaerobic marine [20,51,52] and freshwater [29,53] sediments. Therefore, it is not surprising to find the co-existence of hydrogenotrophic, acetogenotrophic and methylotrophic methanogens in the tropical mangrove sediments in our study. Our findings support the wide distribution of these various methanogens under anaerobic conditions, and also reflect the high diversity of the microbial community and the consequent major metabolic processes, which are likely to contribute to the total methane production in the tropical mangrove sediments. Our study clearly shows that the methylotrophic/acetogenotrophic methanogens prevailed over their hydrogenotrophic counterparts in almost all the samples except SC. This suggests that the methylotrophic methanogens were widespread and could adapt to fluctuating geochemical environments because of their ability to use noncompetitive substrates. The hydrogenotrophic and acetogenotrophic methanogenesis pathways are the most common pathways in soils, and the former usually has a lower production rate than the latter [54]. Therefore, the prevailing numbers of methylotrophic/acetogenotrophic methanogens implies a high methane production in the tropical mangrove sediments, although the *in situ* methane production rate was not measured. In addition, the methylotrophic *Methanococcoides* and *Methanolobus* are major groups in the *Methanosarcinales* order, whereas *Methanosarcina* is less dominant. *Methanosarcina* are known to be predominate at high acetate concentrations [47], therefore, its low abundance in our samples indicates a relatively low acetate level in the mangrove sediments we were investigating.

In our previous study on diazotrophs, we recovered various SRB including *Desulfobotulus*, *Desulfarculus*, *Desulfonatronum* and *Desulfovibrio*, from the mangrove rhizospheres in the same sampling locations we used for this study. We found that they were more abundant in the pristine location at SJ (~40%) than in the most polluted location at SC (~4%) [32]. Their presence indicates not only the potential for bioremediation and the resiliency of the ecosystem to anthropogenic impact, but their coexistence with methanogens in different niches is very likely supported by different substrates. Methanogenesis and sulfate reduction are the terminal steps in the diagenesis of organic carbon [55], and both processes compete for some common substrates, such as hydrogen and acetate. Indeed, in most anaerobic environments, methanogenesis and sulfate reduction are thought to be controlled largely by the amount of available sulfate [56], such that they are usually predominant in low-sulfate freshwater habitats and in sulfate-replete marine environments, respectively. The preference of methanogens for methylated C-1 compounds over hydrogen in marine environments reflects the competition that occurs with SRB. The latter are capable of utilizing hydrogen more efficiently, whereas they are usually unable to use the uncompetitive compounds as substrates [41]. In our previous study, however, SRB were not examined along the depth profile, and so it is not possible to compare the spatial distribution and competition of these two anaerobic groups in each location along the vertical profile with varied substrate composition and concentration. Future investigations using group-specific primers together with the chemistry analysis of sediments at various depths and in different locations would help to elucidate the niche specification of these two groups in tropical mangrove sediments.

### Spatial variation of the methanogens

**Fig 6:**
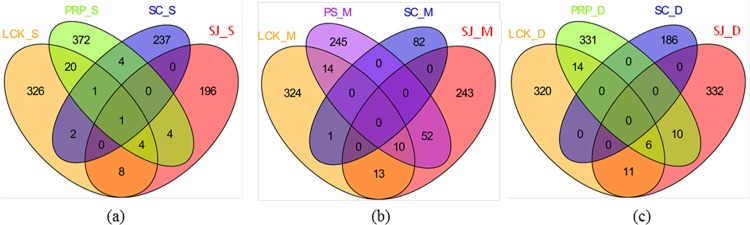
Venn diagrams representing the overlap of OTUs (with 97% sequence similarity as the cutoff value) for (a) surface (_S), (b) middle (_M), and (c) deep (_D) samples collected from the five locations in Singapore.

Venn diagrams were plotted to show the similarities in terms of the overlap of OTUs (at a 97% cutoff value) from the different depths among the five sampling locations (Fig 6). Only one common OTU was shared by all the four surface samples (Fig 6A), whereas samples from the middle and deepest locations had no OTUs in common (Fig 6B and 6C). In the surface samples, the common OTU was identified as being *Methanosarcina spelaei* MC-15 (93% similarity). This is a novel species that has previously been isolated from floating biofilm on a sulphurous subsurface lake in Movile Cave (Mangalia, Romania), and it exhibits autotrophical growth with H_2_/CO_2_, acetate and methanol as well as mono-, di-, tri-methylamine [57]. LCK and PRP shared the highest number of OTUs at the surface (26 OTUs) and deepest (20 OTUs) layers; whereas at the middle depth, PS and SJ had the highest number of shared OTUs (62 OTUs). SC always had fewer OTUs in common with the other sampling sites. In terms of specific unique OTUs, the highest numbers were found at the surface in LCK, PRP and SC, and in the deepest layer at SJ.

**Fig 7:**
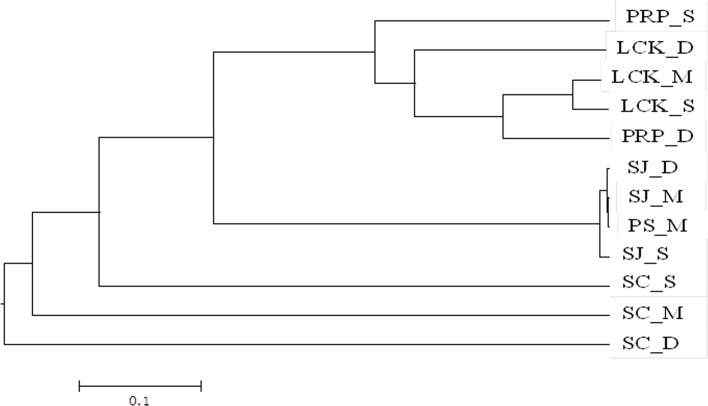
UPGMA clustering of the methanogenic community structures for samples collected from the five locations in Singapore based on total OTUs at the genus level (89% sequence similarity as the cutoff value). Data were square root transformed and the Bray-Curtis similarity was used for clustering analysis.

UPGMA clustering based on the total OTUs at the genus level (89% cutoff value) demonstrated a clear shift in the methanogenic community among the different geographic locations. SJ was clustered with PS, but they were both distinct from the other sampling locations (Fig 7). This is in line with the predominance of *Methanolobus*. Samples from different depths in LCK and PRP were grouped together although with relatively low similarities, as indicated by the long branches. These two locations contained *Methanolobus* and *Methanococcoides*, but at different proportions. All the above locations were only distantly related to SC, where the three depths were predominated by different methanogenic groups (Fig 5).

**Fig 8:**
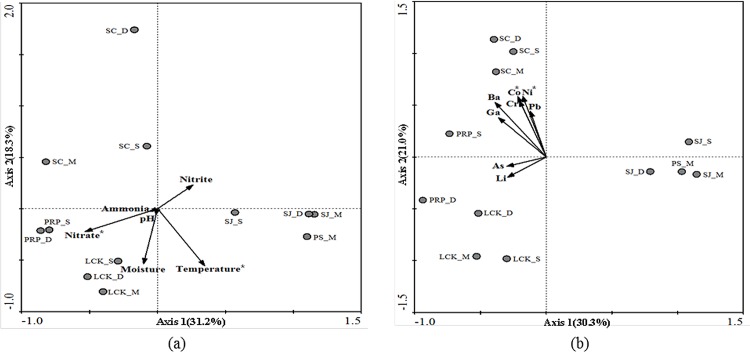
A redundancy analysis (RDA) biplot based on total OTUs (97% sequence similarity as the cutoff value) for samples collected from five locations with (a) environmental parameters and (b) metals as explanatory variables. *p < 0.05.

Multivariate analysis was also performed to show the relationship between the methanogenic community structures (based on the total OTUs at the species level) recovered from the different locations and the associated abiotic factors (Fig 8). A minimum set of abiotic data, determined by the forward selection after removing factors with high collinearity, were included in the RDA analysis. The first two main axes together explain the respective 49.5% (Fig 8A) and 51.3% (Fig 8B) overall variance. Both biplots show that samples from the different depths in SC were located in the upper left panel and were separated from the other samples on Axis 2. Samples from SJ and PS were close to each other in the lower right panel and were distantly located from the other locations on Axis 1. Significance tests of Monte Carlo permutations indicated that temperature, and concentrations of NO_3_^-^, Co and Ni explained most of the spatial variations in the methanogenic communities. A negative correlation was found between NO_3_^-^ and Axis 1 (r = -0.71) and between temperature and Axis 2 (r = -0.82) (Fig 8A), whereas Ni and Co were both positively associated with Axis 2 with similar correlation coefficients (r = 0.86) (Fig 8B). Temperature has been identified as a key factor in the control of methanogenic activity and community composition in sediments [58]. In wetlands at higher latitude, an increase in temperature has been shown to stimulate the growth of methanogens and induce a shift from purely acetoclastic to a combination of acetoclastic and hydrogenotrophic methanogenesis [59]. In our study, the temperature difference along the vertical profile in SC was more obvious than in the other locations, possibly because of the packed texture and lower moisture content of the SC sediments. The distinct temperature discrepancies at the different depths in SC might at least partially explain the clear shift in the types of methanogens with potentially different methanogenic activities. Co and Ni are required for methane-producing reactions via an increase in coenzyme F_430_ and corrinoids [60]; and they are particular important for enzymes catalyzing the methylotrophic pathway [61]. In the multivariate analysis, SC was distributed in the direction of Co and Ni, which is consistent with the highest concentrations of these elements being detected at this location. NO_3_^-^ inhibits methane production by affecting the turnover of both methanogenic precursors (i.e., H_2_ and acetate) and oxidants (sulfate, Fe(III)), and subsequently activating the dinitrifiers, and the sulfate- and iron-reducing bacteria to outcompete the methanogens [62]. LCK had the highest concentration of NO_3_^-^ as a result of the nitrogen fertilizers applied during agricultural activities; however, due to a lack of real-time quantitative data, we are not sure if the abundance of methanogens at this station was significantly lower than that at other stations with lower concentrations of NO_3_^-^.

## Conclusions

Mangrove sediments are important sources of methane production. Therefore, a thorough investigation of the composition of the methanogens residing indifferent depths of mangrove sediments is crucial for understanding the global methane fluxes that occur in the mangrove ecosystems and their contributions to global warming. In this study, by pyrosequencing of the *mcrA* gene, we identified a combination of hydrogenotrophic, acetoclastic and methylotrophic methanogens. The predominance of methylotrophic *Methanosarcinales* at each station reflected the high potential for methane production, possibly with low acetate and high methylated C-1 compounds as the available substrates. The diversity of methanogenic communities at the locations affected by anthropogenic activities was significantly different from that in the pristine SJ. In addition, a decline in the number of potential acetoclastic/methylotrophic methanogens in favor of hydrogenotrophic methanogens was observed along the vertical profile in SC, which was heavily contaminated by heavy metals. UPGMA analysis demonstrated that spatial variations of the methanogenic communities among the different locations were more distinct than those along the vertical profiles at each location. The overall heterogeneity of the methanogenic communities residing in the tropical mangrove sediments could be largely explained by the effect of temperature, as well as the concentrations of NO_3_^-^, Co and Ni. However, whether the anaerobic methanogens present in the shallower layers of sediments (which are potentially oxygenated) are actively involved in methane production or not is still not clear. To further our understanding of the function and activity of methanogens in mangrove sediments, a more detailed survey of the active members and the different substrates they utilize, as well as their associated methane-production rate is required.
